# Thank You to Our Peer Reviewers!

**Published:** 2014-11-01

**Authors:** The Editors of JADPRO

In this issue of the *Journal of the Advanced Practitioner in Oncology* (JADPRO), we are publishing, with our heartfelt thanks, the names of all of the esteemed professionals who completed peer reviews for JADPRO in 2014. As a group, they represent a staggering depth and breadth of experience in specialties and subspecialties within the hematology/oncology field. They provide consistent high-quality, insightful, and rigorous critiques of submitted manuscripts, along with unerring professionalism and good humor.

JADPRO is still a young journal, having just completed our fifth volume, but we are growing and expanding in many exciting ways. It is in a large part because of our peer reviewers that we are able to maintain clinical relevance and scientific quality in all of the articles that we publish.

We hope that during the past year we have made it clear how much we appreciate them and value their indispensable contributions, largely done behind the scenes. But perhaps by publishing the names of all of the 2014 JADPRO peer reviewers here, they will receive the recognition they deserve for stellar work.

If you would like to get involved as a peer reviewer for JADPRO, please don’t hesitate to contact us at editor@advancedpractitioner.com to let us know about your interests and areas of expertise.

—From the Editors of *JADPRO*

**Paula Anastasia**, RN, MN, AOCN®

**Andria N. Arrington**, PA-C

**Karen A. Beaty**, PA-C

**Catherine S. Bishop**, DNP, NP, AOCNP®

**Karyl Blaseg**, RN, MSN, OCN®

**Tami Borneman**, RN, MSN, CMS

**Jeannine M. Brant**, PhD, APRN, AOCN®

**Matthew M. Burke**, MBA, RN, MSN, APRN-BC

**Christopher J. Campen**, PharmD, BCPS, BCOP

**Amber B. Clemmons**, PharmD, BCOP

**Kathleen N. Clifford**, RN, MSN, FNP-BC, AOCNP®

**Lorinda Coombs**, MSN, FNP-BC, AOCNP®

**Colleen Cuthbert**, MN, RN, NP

**Carrie F. Daly**, RN, MS, APN®

**Deena Damsky Dell**, MSN, RN-BC, AOCN®

**Kathy Diener Dasse**, PharmD, BCOP

**Mary Caroline Deigert**, BS, MPAS, PA-C

**Beth Eaby-Sandy**, MSN, CRNP, OCN®

**Denice Economou**, RN, MN, CNS, AOCN®, CHPN

**Jessica M. Engel**, RN, RNP-BC, AOCNP®

**Beth Faiman**, PhD(c), MSN, APN-BC, AOCN®

**Theresa Wicklin Gillespie**, PhD, MA

**Paige M. Goforth**, MMS, PA-C

**Sherry Goldman**, NP, RN, CBCN

**Meaghan McEntee Gomez**, ANP-BC, AOCNP®

**Clara Granda-Cameron**, DNP, CRNP, AOCN®

**Carolyn Grande**, CRNP, AOCNP®

**Kristina M. Gregory**, RN, MSN, OCN®

**Marilyn Haas-Haseman**, PhD, ANP-BC

**Jacqueline Hale**, RN, APN, AOCN®

**Heather Hampel**, MS, LGC

**Megan J. Holder**, APN

**Heather M. Hylton**, MS, PA-C

**Lisa Kottschade**, RN, MSN, CNP

**Kristen Kreamer**, CRNP, MSN, AOCN®, APRN, BC

**Gail Kwarciany**, RN, MSN, BC, OCN®, AOCNS®

**Sandra E. Kurtin**, RN, MS, AOCN®, ANP-C

**Jessica Latchman**, MSN, ARNP, AOCNP®

**Joanne Lester**, PharmD, CRNP, ANP-BC, AOCN®

**Lydia T. Madsen**, PharmD, RN, AOCNS®

**Steve Malangone**, MSN, FNP-C

**Kelley D. Mayden**, MSN, FNP, AOCNP®

**Erin McMenamin**, MSN, CRNP, ANP-BC, AOCN®

**Lynora Metoyer**, RN, MSN, FNP

**Ali McBride**, PharmD, MS, BCPS

**Suzanne McGettigan**, MSN, CRNP, AOCN®

**Cheryl J. McGovern**, PA-C

**Joseph B. Narus**, DNP, GNP-BC, ANP

**Susan Newton**, RN, MS, AOCN®, AOCNS®

**Nancy M. Nix**, PharmD, BCPS, BCOP

**Patricia Palmer**, RN, MS, AOCNS®

**Lisa Parks**, MS, RN, CNP, ANP-BC

**Jody Pelusi**, PhD, FNP, AOCNP®

**Derek Peterson**, PharmD

**Todd Pickard**, MMSC, PA-C

**Christopher A. Puleo**, PA-C

**Karen Roesser**, RN, MS, AOCN®

**Sandra Rome**, RN, MN, AOCN®

**Jean Rosiak**, DNP, RN, ANP-BC, AOCNP®, CBCN

**Meghan Routt**, MSN, RN, GNP/ANP, AOCNP®

**Leanne M. Sakamoto**, PharmD

**Brenda K. Shelton**, DNP, RN, CCRN, AOCN®

**Ellen M. Lavoie Smith**, PhD, APN-BC, AOCN®

**Wendy J. Smith**, ACNP, AOCN®

**Robin M. Sommers**, DNP, ANP-BC, AOCNP®

**Suzanne Swain-Cabriales**, RN, OCN®, CCRP

**Eric D. Tetzlaff**, MHS, PA-C

**Timothy Tyler**, PharmD, FCSHP

**Carol S. Viele**, RN, MS, CNS, OCN®

**Constance Visovsky**, PhD, RN, ACNP-BC

**Wendy H. Vogel**, MSN, FNP, AOCNP®

**Suzanne Walker**, CRNP, MSN, AOCN®

**Dwanna Ward-Boahen**, DNP, NP-C, AOCNP®

**Steven H. Wei**, MS, MPH, PA-C

**Rita Wickham**, PhD, RN, AOCN®

**Tara C. Wray**, RN, MSN, APN-BC

**James Young**, RPH

**Laura Zitella**, MS, RN, ACNP-BC, AOCN®

